# *Vicia faba*-PGPB association improves soil health as a sustainable strategy to remediate moderately Pb and Cd contaminated soils

**DOI:** 10.1371/journal.pone.0353746

**Published:** 2026-07-15

**Authors:** Omar Saadani, Souhir Abdelkrim, Wael Taamalli, Imen Challougui Fatnassi, Khedhiri Mannai, Moez Jebara, Salwa Harzalli Jebara

**Affiliations:** 1 Laboratory of Legumes and Associated Agro-systems, Center of Biotechnology of Borj Cedria, Hammam Lif, Tunisia; 2 Faculty of Sciences of Tunis, University Tunis El Manar, CP.2092, El-Manar Tunis, Tunisia; 3 Laboratory of Olive Biotechnology, Center of Biotechnology of Borj Cedria, Hammam Lif, Tunisia; 4 Higher Institute of Biotechnology of Beja, University of Jendouba, Beja, Tunisia; Universidade de Coimbra, PORTUGAL

## Abstract

Phytoremediation is an eco-friendly strategy for heavy metal bioremediation. This study focuses on assessing the potential of faba bean- plant growth promoting bacteria symbiosis in phytoremediation and soil fertility improvement of HMs contaminated soils. *Vicia faba* L. var. *minor* Saber 02 was inoculated with a consortium of three efficient and HMs resistant PGPB (*Rhizobium* sp. CCNWSX0481, R. *leguminosarum* bv. *viciae* and *Pseudomonas* sp.) and cultivated in soil treated with Cd and Pb to establish three contamination levels: uncontaminated (S1), moderately contaminated (S2; 2 mg kg^-1^ Cd and 100 mg kg^-1^ Pb), and highly contaminated (S3; 4 mg kg^-1^ Cd and 200 mg kg^-1^ Pb). Bacterial inoculation enhanced plant growth and metal uptake, most significantly in the moderately contaminated soil (S2). An increase in shoot dry weight and nodule dry weight was observed after bacterial inoculation mostly in the moderately contaminated soil S2. Furthermore, the effect of bacterial inoculation was particularly pronounced in S2 soil, resulting in significant increases in Pb and Cd accumulation in the shoots by 66% and 441%, respectively, compared to the uninoculated plants. Similarly, inoculated plants grown in S2 soil exhibited substantially higher total heavy metal contents than the uninoculated plants, reaching 179% for Pb and 319% for Cd, respectively. This increase was associated with an enhancement in the concentration of non-protein thiols, particularly in S2 soil, where inoculation increased root NPT levels by 49% compared to the uninoculated plants. Nevertheless, HMs induced a significant increase in roots enzyme such as superoxide dismutase, catalase and glutathione reductase. The inoculation further enhancing their activities essentially in S2. Moreover, PGPB considerably reduced total Pb as well as both the total and available fractions of Cd, mainly in S2 soil and increased total nitrogen and available phosphorus content, urease and β-glucosidase activities. The obtained results highlight the effectiveness of *V. faba* L var. *minor* Saber 02- PGPB symbiosis in the reclamation of moderately Pb and Cd contaminated soils. The bacterial consortium could be used as biofertilizer to improve soil quality of Cd/Pb contaminated sites.

## Introduction

Heavy metals (HMs) soil contamination is an important environmental problem caused by industrialization, intensive agriculture and extensive mining; they induce serious damage on the ecosystem due to their inability to be degraded and cause a serious threat to plants, microorganisms, animals and humans through the food chain [1].

Lead (Pb) is one of the most common HMs contaminants in the environment and plants are exceedingly harmful to it; it has low solubility and is classified as both carcinogenic and mutagenic [2]. Cadmium (Cd) prevailing in most of the agricultural lands of the world, causes adverse effects on living organisms including higher plants, as numerous studies affirmed that it triggers phytotoxicity in plants [3,4].

Remediation of HMs contaminated soils by physico-chemical methods is expensive and impracticable, so it is more efficient to remove or reduce HMs contamination by bioremediation processes which use biological mechanisms to control hazardous contaminants through microorganisms and plants [5–8]. Phytoremediation is a method of bioremediation process using plants for treatment of polluted soils, in this context, legumes-rhizobia symbiosis become a natural choice when searching for plants that can grow on contaminated soils with poor nutritional values. Interestingly, this eco-friendly symbiosis is one of the beneficial plant-microbe interactions that constitute an elite group for novel and effective soil restoration [9,10]. Recently, scientists have been interested in nitrogen-ﬁxing microbes as a type of plant growth-promoting bacteria (PGPB) in symbiosis with legumes for phytoremediation of contaminated soils [11–13].

Furthermore, this symbiosis could be a good biofertilizer that enhances soil fertility by enhancing P and N bioavailability, since these symbiotic microorganisms are qualified by plant growth promoting (PGP) traits such as nitrogen fixation, phosphorus solubilization, siderophore and phytohormone production such as indole-3-acetic acid (IAA), cytokinins [14–16]. However, the response of legume-PGPB to HMs contamination depends on metal characteristics, including its solubility, bioavailability and the physiochemical process of soil.

On the other hand, heavy metals induced phytotoxicity triggers higher level of reactive oxygen species (ROS) and the antioxidant pathway constitutes an important adaptive response to mitigate stress. The antioxidative defence machinery of plants is formed by enzymatic systems including superoxide dismutase (SOD), glutathione reductase (GR), ascorbate peroxidase (APX), peroxidase (POX) and catalase (CAT); the non-enzymatic anti-oxidative systems include essentially glutathione (GSH), proline, ascorbate (AsA) and carotenoids [17,18]. Nevertheless, it has been demonstrated that PGPB inoculation can have a positive effect on the biochemical responses of legumes- soil systems in HMs contaminated sites and becomes a promising strategy for the phytomanagement of polluted soils [9,14].

Faba bean (*Vicia faba* L.) is an efficient nitrogen-fixing legume that enhances soil fertility through nitrogen-rich residues and is well known for its rapid growth and high biomass production. It also has the capacity to accumulate and translocate heavy metals such as Cd and Pb in different plant tissues [19]. However, the effects of PGPB on metal uptake and biochemical responses of the *Vicia faba*–soil system remain poorly understood, particularly under varying contamination levels. Therefore, evaluating *Vicia faba*–PGPB symbiosis under moderate and high heavy metal stress is essential to optimize phytoremediation and enhance soil fertility in contaminated environments. Therefore, investigating the *Vicia faba*–PGPB symbiosis under moderate and high heavy metal stress is essential. Such an approach can provide new insights into optimizing phytoremediation efficiency while simultaneously improving soil fertility and supporting sustainable management of contaminated soils.

In the present work, *V. faba* was inoculated with three bacterial strains selected from a collection of soil bacteria isolated from nodules of *V. faba* grown in heavy metal-contaminated soils. The strains, *Rhizobium* sp. CCNWSX0481, *R. leguminosarum* bv. *viciae*, and *Pseudomonas* sp., were chosen based on their efficiency and tolerance to heavy metals [20]. Their ability to produce indoleacetic acid (IAA) and siderophores, as well as their potential for phosphate solubilization, has already been demonstrated by Saadani et al. [21].

Here we explore the performance of the used symbiosis in phytoremediation and recovery of contaminated sites. Consequently, the intentions of current research were: *(i)* to assess the potentialities of *V. faba* L var.. *minor* Saber 02 -PGPB symbiosis in phytoremediation of moderately and highly Pb and Cd contaminated soils, *(ii)* to investigate the contribution of PGPB inoculation in plant mechanisms responses involved in mitigation of the HMs induced stress in *V. Faba*, *(iii)* to assess changes in the soil in terms of total and available metal fractions as well as soil fertility and quality.

## Materials and methods

### Soil sampling

Soil samples used for the cultivation of *Vicia faba* L. var. *Mino* Saber 02 were taken from 0–25 cm depth of an agricultural field located in northern Tunisia. Sampling was conducted with prior authorization from the owner of the location. The soil was classified as sandy loam (10% clay, 7% silt, 83% sand). The soil properties were as follows: pH 8.44; 0.62% organic matter; 39.2 ppm of available phosphorus; 0.31% total nitrogen; 12.3 mg kg^− 1^ Pb and 0.5 mg kg^− 1^ Cd (Table 1).

**Table 1:** Characterization of the soil used for plant cultivation.

pH	8.44 ± 0.25
OM (%)	0.62 ± 0.06
N_tot_ (‰)	0.31 ± 0.02
P_2_O_5_ (ppm)	39.20 ± 2.04
Pb (mg kg^-1^)	12.31 ± 0.75
Cd (mg kg^-1^)	0.5 ± 0.09

Data are means ± standard deviation (n=3). OM, organic

matter; Ntot, total nitrogen, P_2_O_5_, available phosphorus

### Pot experiment and soil contamination

A greenhouse pot experiment was conducted, for this purpose, 15 kg of air dried, homogenized and sieved soil samples were transferred in pots and mixed thoroughly with different concentrations of CdCl_2_ and PbCl_2_ solutions. Heavy metal concentrations used in the experiment were (mg kg^-1^): 0; 2 and 4 for Cd; 0; 100 and 200 for Pb; hereafter termed S1 (without metal addition), S2 (moderately contaminated) and S3 (highly contaminated), compared WHO (World Health Organization) permissible limits of heavy metals in soil [22,23].

### Plant cultivation and bacterial inoculation

Seeds of *V. faba* (Saber 02 variety) were sterilized with 70% ethanol and 0.2% mercuric chloride (HgCl_2_) then rinsed with distilled water. Seeds were germinated on 0.9% agar at 25 °C in continuous darkness for 3days.

Four uniformly germinated seedlings were initially transplanted per pot to ensure successful establishment. After emergence, each pot was thinned to a single seedling. The individual pot, containing one plant, thus constituted the experimental unit, and all measurements were performed directly on that plant.

The experiment followed a completely randomized design with a two-way factorial structure comprising two fixed factors: inoculation (IN; uninoculated control vs. bacterial inoculation) and soil contamination (SC; three levels: S1, S2, and S3), yielding six treatment combinations each replicated three times (n = 3 pots per combination). Pots were arranged in a completely randomized layout within the greenhouse and repositioned weekly to minimise spatial variability.

Plant inoculation was performed by the bacterial consortium formed by bacterial strains previously isolated from root nodules of *V. faba* L. var. *minor* Saber 02 cultivated in heavy metal contaminated soils and characterized as *Rhizobium* sp. CCNWSX0481, *R. leguminosarum* bv. *viciae* and *Pseudomonas* sp. [20]. The inoculation was made as described by Abdelkrim et al. [24]. Legumes were harvested at the flowering stage.

### Soil and plant nitrogen and phosphorus content

Plant and soil nitrogen were determined according to Kjeldahl [25].

Phosphorus concentration in plant tissue was determined using 30 mg of dried material. Total P was extracted by acid digestion with 5.5 M H₂SO₄ in the presence of potassium persulfate (0.13 M) at 120 °C for 30 min [26].

### Soil pH, carbon and organic matter content

To determine carbon content, 0.5 g of soil sample was treated with 96% H_2_SO_4_ in the presence of 5 ml of potassium dichromate (K_2_Cr_2_O_7_) solution0.27 M at 135°C for 30 min. The mixture was water diluted to final volume of 100 ml and stabilized for one hour. Supernatant was centrifuged for 10 min at 2000 g then carbon was determined colorimetrically against standard glucose at 585 nm. Soil organic matter was measured by the gravimetric loss after 4 h aching of 20 g of soil samples at 525 °C. The pH of the soil was determined by means of a calibrated pH meter by preparing 1: 2.5 (w:v) soil: water suspension.

### Determination of *V. faba* L. var. *minor* Saber 02 heavy metal accumulation

Heavy metal accumulation in shoots and roots was determined in 0.5 g of dried plant material digested by a mixture of nitric acid (HNO_3_) and perchloric acid (4:1, v/v) until dryness at 100°C for 2 h and diluted in 20 ml HNO_3_. Heavy metal concentrations were determined by atomic absorption spectroscopy (Perkin Elmer, model AAnalyst 700) and total HMs extracted and accumulated by *V. faba* L. var. *minor* Saber 02 was measured by adding shoot and root concentrations and multiplying them by their respective dry weights:


$$\begin{array}{c} \text{Total HM }({\text{mg plant}}^{-\text{1}})\;=\;(\text{shoot HM }({\text{mg kg}}^{-\text{1}})\;+\text{ root HM }({\text{mg kg}}^{-\text{1}})\;*\text{ total plant dry Weight }(\text{kg}) \end{array}$$

Translocation factor (TF) was determined according to Ahmad et al. [27], TF indicates ratio of metal in plant shoots to roots.

### Determination of total extractable heavy metals

Soil Pb and Cd available fractions were extracted by the treatment of soil samples (2 g) by 1 M of ammonium acetate (C_2_H_7_NO_2_) solution and 0.2 M EDTA (ethylene diamine tetra-acetic acid) Lakanen and Erviö [28]. Total metals were determined by digesting soil samples (0.5 g) in a mixture of 37% HCl and 63% HNO3 (3/1: v/v) at 100 °C. The obtained suspension was diluted with 0.6% HNO_3_ and the mixture was filtered using Whatman filter paper before analysis. Trace element concentrations were determined by atomic absorption spectroscopy (Perkin Elmer, model AAnalyst 700).

### Soil enzyme activities

#### Urease activity.

For the determination of urease activity 1g of the soil was incubated for 2 h at 37°C with borate buffer (pH10) and 0.5 ml urea (0.48%). Released ammonium was extracted and filtered using potassium chloride (KCl) 1 M and hydrochloric acid (HCl) 10 mM, incubated 30 min at 25°C, reaction was measured calorimetrically at 690 nm by adding 3 ml of filtrate to 5 ml of sodium salicylate (17%) and 2 ml dichloroisocyanurate (0.1%), ammonium chloride (NH_4_Cl) solution was used as standard [29].

#### *β*-glucosidase.

For the *β*-glucosidase activity assay, 0.5 g of soil was incubated for 90 min at 37 °C, with 1.25 ml phosphate buffer (0.1 M, pH6) and 0.5 ml 4-nitrofenyl-*β*-D-glucopyranoside (25 mM). The mixture was centrifuged 5 min at 4000 rpm and the p-nitrophenol was measured spectrophotometrically at 405 nm [30].

### Plant biochemical analysis

#### Total chlorophyll.

Fresh leaves (0.1 g) were cut into pieces, homogenized in 80% acetone and left in darkness at 4 °C for 72 h. The absorbance of the mixture was measured at 470, 649 and 665 nm and pigment concentration determined according to the formula of Lichtenthaler and Wellburn [31].

### Lipid peroxidation

The level of lipid peroxidation was determined in terms of malondialdehyde (MDA) concentration as reported by Chen et al*.* [32]:

$\text{MDA }(\mu \text{M})\;=\;\text{6}.\text{45 }*\;({\text{A}}_{\text{532}}\;-{\text{A}}_{\text{600}})\;-\;\text{0}.\text{56 }*{\text{A}}_{\text{4}\text{5}\text{0}}\;(\text{A}:\text{ sample absorbance}).$

### Measurement of antioxidant enzyme activities in plants

#### Total protein extraction and determination.

The protein concentration in fresh weight was determined according Bradford [33] using bovine serum albumin as standard.

### Enzyme activities

Superoxide dismutase SOD, ascorbate peroxidase APX, guaiacol peroxidase GPOX and catalase CAT activities were assayed as previously described by Jebara et al*.* [34]. Glutathione reductase GR (EC 1.6.4.2) activity consists in the reduction of oxidized glutathione (GSSH) toits reduced form (GSH). The enzyme activity was measured following the spectrometric absorption at 340 nm and calculated using the extinction coefficient ε = 6.22 mM^-1^ cm^-1^ [35].

### Measurement of non-proteinthiols (NPT)

Non-protein thiols content was calculated using the extinction coefficient of DTNB after spectrophotometric measurement at 412 nm [36].

### Statistical analysis

For each response variable, a fixed-effects model was fitted using a generalized linear model (GLM) with a Gaussian distribution and identity link. Significance of model terms was assessed using analysis of variance with F-tests. Model assumptions were evaluated using simulation-based DHARMa residual diagnostics, including tests for dispersion, residual uniformity, and outliers (α = 0.05). Across all variables, these diagnostics were non-significant, indicating no evidence of model misspecification, heteroscedasticity or influential outliers. When the IN × SC interaction was significant, all six treatment combinations were compared using Tukey’s honestly significant difference (HSD) test at α = 0.05 based on estimated marginal means. When the interaction was not significant, the model was refitted without the interaction term to allow interpretation of main effects. Post hoc comparisons were conducted only for significant factors: soil contamination effects were denoted by uppercase bold letters (**A-C**), consistent across inoculation levels to reflect marginal means, whereas inoculation effects were indicated by lowercase italic letters (*a*-*b*). Shoot GPOX activity included left-censored observations in the uninoculated S2 and S3 treatments. A Tobit censored regression model was initially considered but proved unsuitable, as complete separation in the likelihood surface produced degenerate parameter estimates. The data were therefore recoded as a binary response (detected vs. not detected) and analyzed using Fisher’s Exact Test.

All analyses were conducted in R v. 4.5.1 [37], using the packages *emmeans* [38], *multcomp* [39], *DHARMa* [40] and *car* [41]. Figures were produced using *ggplot2* [42] and *patchwork* [43].

## Results

To evaluate the effects of inoculation and contamination, a two-way analysis of variance was conducted with inoculation treatment (IN) and soil contamination (SC) as the main factors (Table 2). Results revealed significant effects of the interaction (IN × SC) on SDW, NDW, N content in shoots and roots, P content in roots, MDA content in roots, the activities of SOD, CAT and GR in both shoots and roots, APX activity in shoots, Pb and Cd accumulation in shoots, Cd accumulation in roots, total Pb and Cd content in plants, translocation factors of Pb and Cd, soil total N content, soil β-glucosidase and urease activities, total Pb and total Cd in soil, and extractable Pb and Cd in soil, indicating that the response to inoculation depended on the contamination level. RDW, shoot P content, total chlorophyll, NPT in roots, GPOX root activity, root Pb content, and soil available P were significantly affected by both IN and SC independently. Shoot MDA and shoot NPT were affected only by SC, with no significant effect of IN. APX root activity showed no significant effect of either factor. For GPOX activity in shoots, the Fisher’s Exact Test on detection frequencies confirmed that inoculation had a significant effect (*p* = 0.0019) on maintaining the GPOX defence system across the contamination gradient.

**Table 2:** Summary of two-way ANOVA testing the effects of inoculation (IN), soil contamination (SC), and their interaction (IN × SC) on measured variables.

Variable	IN (*p*)	SC (*p*)	IN x SC (*p*)
SDW	< 0.001	< 0.001	**< 0.001**
RDW	**< 0.001**	**0.001**	0.064
NDW	< 0.001	< 0.001	**< 0.001**
N shoot	< 0.001	< 0.001	**< 0.001**
N root	< 0.001	< 0.001	**< 0.001**
P shoot	**< 0.001**	**< 0.001**	0.075
P root	< 0.001	< 0.001	**< 0.001**
Total chlorophyll	**< 0.001**	**< 0.001**	0.131
MDA shoot	0.252	**0.002**	0.075
MDA root	< 0.001	< 0.001	**0.001**
NPT shoot	0.126	**< 0.001**	0.054
NPT root	**< 0.001**	**< 0.001**	0.153
SOD shoot	< 0.001	< 0.001	**< 0.001**
SOD root	< 0.001	< 0.001	**< 0.001**
CAT shoot	< 0.001	0.017	**0.030**
CAT root	< 0.001	< 0.001	**< 0.001**
GPOX root	**< 0.001**	**0.005**	0.124
APX shoot	0.004	0.001	**< 0.001**
APX root	0.610	0.121	0.247
GR shoot	< 0.001	< 0.001	**0.001**
GR root	< 0.001	0.002	**< 0.001**
Pb shoot	0.017	< 0.001	**< 0.001**
Pb root	**0.013**	**< 0.001**	0.170
Cd shoot	< 0.001	< 0.001	**< 0.001**
Cd root	0.226	< 0.001	**< 0.001**
Total Pb content	< 0.001	< 0.001	**< 0.001**
Total Cd content	< 0.001	< 0.001	**< 0.001**
Total N	< 0.001	< 0.001	**0.005**
P_2_O_5_	**< 0.001**	**< 0.001**	0.067
*β*-Glucosidase	0.125	0.007	**0.002**
Urease	< 0.001	< 0.001	**< 0.001**
Total Pb	< 0.001	< 0.001	**0.035**
Total Cd	< 0.001	< 0.001	**0.003**
Extractable Pb	< 0.001	< 0.001	**< 0.001**
Extractable Cd	< 0.001	< 0.001	**< 0.001**
TF of Cd	< 0.001	< 0.001	**< 0.001**
TF of Pb	< 0.001	< 0.001	**< 0.001**

SDW, shoot dry weight; RDW, root dry weight; NDW, nodule dry weight; P, phosphorus; N, nitrogen; MDA, malondialdehyde; NPT, non-protein thiols content; SOD, CAT, GPOX, APX and GR, antioxidant enzyme activities; Pb and Cd, accumulation in shoots and roots; Total Pb and Cd, content in plants; Total N, Soil total nitrogen content; P_2_O_5_, soil available phosphorus; Total Pb and Cd in soils. NE = not estimable.

### Effect of inoculation by PGPB on plant agronomic parameters of *V. faba* L. var. *minor* Saber 02 cultivated in heavy metals contaminated soils

The interaction (IN × SC) showed significant effects on shoot dry weight (SDW) and nodule dry weight (NDW) (Table 2). Inoculation proved most beneficial in the moderately contaminated S2 soil, where it effectively more than doubled SDW (from 0.79 to 1.81 g) and increased NDW 4-fold compared to uninoculated controls (Table 3). However, in the highly contaminated S3 soil, these growth parameters showed no significant differences between inoculated and uninoculated plants. In contrast, root dry weight (RDW) was significantly influenced by the independent effects of both factors. Comparing the levels of the main effects reveals that inoculation consistently enhanced root biomass while soil contamination was characterized by a significant reduction in root development in S3 compared to S2.

**Table 3:** Effects of soil contamination and plant inoculation on shoot dry weight (SDW), root dry weight (RDW), nodule dry weight (NDW), phosphorus (P), nitrogen (N), chlorophyll, malondialdehyde (MDA), non-protein thiols (NPT) content and antioxidant enzyme activities in shoots and roots of *V. faba* L. var. *minor* Saber 02.

		Uninoculated			Inoculated		
		S1	S2	S3	S1	S2	S3
SDW (g plant^-1^)		1.48 ± 0.15^a^	0.79 ± 0.18^b^	0.40 ± 0.13^b^	1.52 ± 0.26^a^	1.81 ± 0.22^a^	0.69 ± 0.25^b^
RDW (g plant^-1^)		0.25 ± 0.09^**AB***b*^	0.24 ± 0.03^**A***b*^	0.09 ± 0.03^**B***b*^	0.33 ± 0.09^**AB***a*^	0.58 ± 0.22^**A***a*^	0.27 ± 0.13^**B***a*^
NDW (mg plant^-1^)		56.7 ± 8.5^ab^	15.1 ± 5.3^c^	3.6 ± 2.8^c^	43.8 ± 6.4^b^	63.8 ± 15.9^a^	11.6 ± 2.6^c^
N content (%)	Shoot	5.13 ± 0.10^c^	4.67 ± 0.09^d^	2.33 ± 0.05^e^	6.53 ± 0.13^b^	7.01 ± 0.14^a^	4.59 ± 0.11^d^
	Root	5.13 ± 0.10^b^	3.73 ± 0.07^e^	1.40 ± 0.03^f^	5.59 ± 0.11^a^	4.67 ± 0.09^c^	4.20 ± 0.08^d^
P content (%)	Shoot	0.67 ± 0.07^**B***b*^	0.7 ± 0.07^**A***b*^	0.32 ± 0.05^**C***b*^	0.87 ± 0.02^**B***a*^	1.01 ± 0.01^**A***a*^	0.50 ± 0.03^**C***a*^
	Root	0.17 ± 0.006^b^	0.10 ± 0.003^c^	0.05 ± 0.003^c^	0.20 ± 0.04^b^	0.34 ± 0.04^a^	0.07 ± 0.006^c^
Total chlorophyll (mg g^-1^ FW)		1.05 ± 0.06^**A***b*^	1.11 ± 0.17^**A***b*^	0.69 ± 0.13^**B***b*^	1.27 ± 0.05^**A***a*^	1.21 ± 0.01^**A***a*^	1.04 ± 0.09^**B***a*^
MDA (µmol g^-1^ FW)	Shoot	51.5 ± 3.4^**B**^	44.1 ± 11.8^**B**^	53.9 ± 2.9^**A**^	47.2 ± 5.5^**B**^	45.7 ± 1.3^**B**^	66.3 ± 2.3^**A**^
	Root	14.6 ± 3.6^b^	27.8 ± 3.6^a^	29.9 ± 0.6^a^	8.7 ± 0.3^b^	8.6 ± 4.1^b^	10.5 ± 0.9^b^
NPT (mM g^-1^ FW)	Shoot	458 ± 64.7^**A**^	141 ± 17.1^**C**^	237 ± 1.1^**B**^	381 ± 19.5^**A**^	151 ± 19.1^**C**^	235 ± 10.6^**B**^
	Root	40.4 ± 11.5^**B***b*^	55.8 ± 4.2^**A***b*^	44.7 ± 2.8^**B***b*^	55.8 ± 10.1^**B***a*^	83.3 ± 5.3 ^**A***a*^	53.9 ± 8.3^**B***a*^
SOD (USOD µg^-1^ P)	Shoot	0.07 ± 0.003^c^	0.10 ± 0.007^b^	0.13 ± 0.003^a^	0.05 ± 0.007^d^	0.06 ± 0.001^d^	0.07 ± 0.001^d^
	Root	0.15 ± 0.003^d^	0.22 ± 0.02^c^	0.34 ± 0.03^b^	0.38 ± 0.006^ab^	0.39 ± 0.008^a^	0.37 ± 0.003^ab^
CAT (µM H_2_0_2_ min^-1^ µg^-1^ P)	Shoot	0.25 ± 0.06^ab^	0.22 ± 0.02^bc^	0.33 ± 0.03^a^	0.14 ± 0.005^c^	0.21 ± 0.06^bc^	0.20 ± 0.02^bc^
	Root	0.69 ± 0.11^d^	0.66 ± 0.05^d^	2.46 ± 0.13^a^	1.73 ± 0.15^b^	2.64 ± 0.15^a^	1.01 ± 0.09^c^
GPOX (nM H_2_0_2_ min^-1^ µg^-1^ P)	Shoot	0.06 ± 0.001^**A**^	ND^**AB**^	ND^**B**^	0.01 ± 0.001^**A**^	0.03 ± 0.007^**AB**^	0.01 ± 0.002^**B**^
	Root	0.14 ± 0.02^**B***b*^	0.25 ± 0.02^**A***b*^	0.26 ± 0.07^**B***b*^	0.38 ± 0.06^**B***a*^	0.51 ± 0.08^**A***a*^	0.4 ± 0.004^**B***a*^
APX (nM ascorbate min^-1^ µg^-1^ P)	Shoot	0.97 ± 0.17 ^c^	2.52 ± 0.44 ^a^	1.05 ± 0.32^bc^	0.42 ± 0.11^c^	0.61 ± 0.12^c^	1.88 ± 0.54^ab^
	Root	8.13 ± 1.62	7.67 ± 1.36	5.77 ± 1.85	8.85 ± 1.97	6.35 ± 1.03	7.49 ± 0.95
GR (nM NADPH min^-1^ µg^-1^ P)	Shoot	0.27 ± 0.14^b^	1.53 ± 0.41^a^	1.02 ± 0.08^a^	0.17 ± 0.04^b^	0.38 ± 0.18^b^	0.07 ± 0.01^b^
	Root	1.08 ± 0.46^c^	4.53 ± 0.60^b^	3.92 ± 0.70^b^	6.95 ± 0.37^a^	6.60 ± 0.30^a^	6.59 ± 1.05^a^

Data are means ± standard deviation (n = 3). Values are mean ± SD (n = 3). When the interaction was significant (p < 0.05), letters compare all treatment combinations by Tukey HSD (α = 0.05). When interaction was NS, uppercase bold letters compare soil levels and lowercase italic letters compare inoculation treatments (Tukey α = 0.05). Within each line, means followed by a common letter are not significantly different at *p* < 0.05 according to Tukey’s HSD-test. Cells without letters: no significant effect detected. ND: Not Detected. SDW, shoot dry weight; RDW, root dry weight; NDW, nodule dry weight; P, phosphorus; N, nitrogen; MDA, malondialdehyde; NPT, non-protein thiols content; SOD, CAT, GPOX, APX and GR, antioxidant enzyme activities.

Regarding nutrient accumulation, the significant interaction for both shoot and root Nitrogen (N) content reveals that the bacterial consortium’s impact on nitrogen nutrition differed across contamination levels. In uninoculated plants, heavy metal stress induced a severe and progressive depletion of N, with concentrations falling to their lowest in S3 (2.33% in shoots and 1.40% in roots). In contrast, inoculation successfully reversed this trend in S2, where shoot N reached its peak value of 7.01%, a 50% increase compared to uninoculated S2 plants. While N levels declined in S3 for all treatments, the inoculated plants maintained significantly higher N (4.59% in shoots and 4.20% in roots), representing a 2-fold and 3-fold higher concentration, respectively, than their uninoculated counterparts.

A similar interaction effect was observed for root phosphorus (P) content, where inoculation specifically triggered a 3-fold increase in P levels in S2 soil compared to the uninoculated control (Table 3). In the shoots, the main effects were highly determinant. Inoculation significantly and independently enhanced shoot P content, while soil contamination caused a modest increase from S1 to S2 followed by a sharp reduction in S3, regardless of the inoculation status (Table 3).

### Effect of inoculation by PGPB on malondialdehyde, chlorophyll and non-protein thiols content in *V. faba* L. var. *minor* Saber 02 cultivated in heavy metals contaminated soils

Regarding MDA content in the roots, a significant interaction indicated that the efficacy of inoculation was dependent on the contamination level. In uninoculated plants, heavy metal stress in S2 and S3 soils significantly increased lipid peroxidation, with MDA levels rising by 91% and 105%, respectively, compared to the S1 control. Conversely, MDA levels in inoculated roots remained low and stable across all soil types (not significantly different from the uninoculated S1 control), corresponding to approximately 69% and 65% lower MDA in S2 and S3, respectively, compared to their uninoculated controls. In the shoots, only the highly contaminated S3 soil significantly increased MDA accumulation, regardless of the inoculation status.

Regarding NPT levels, they were more pronounced in the shoots than in the roots, essentially in *V. faba* L. var. *minor* Saber 02 cultivated in S1 (Table 3). In the roots, both SC and IN significantly and independently influenced NPT levels.

Inoculation significantly enhanced the root thiol pool across all treatments, with inoculated plants maintaining a higher mean concentration (64.3 mM g^-1^ FW) compared to uninoculated plants (47 mM g^-1^ FW) while SC increased root NPT levels in S2 (Table 3). In the shoots, however, only the effect of soil contamination was significant thereby decreasing NPT content in S2 and to lesser degree in S3.

For chlorophyll content, both IN and SC exerted significant independent effect (Table 2). Comparing the levels of soil contamination showed that S3 induced a significant decrease in chlorophyll pigments (averaging a 26% reduction across both IN levels) compared to S1. Simultaneously, inoculation resulted in a significant increase in chlorophyll rates, shifting the mean from 0.95 mg g^-1^ FW in uninoculated plants to 1.17 mg g^-1^ FW in inoculated ones across all soil treatments (Table 3).

### Antioxidant enzyme activities

The antioxidant enzyme activities were also determined to understand their role in metallic stress tolerance. Regarding root antioxidant responses, SOD, GR and CAT activities were predominantly determined by a significant interaction between factors. Inoculation significantly elevated SOD activity in S1 and S2, while no significant difference between treatments was observed in S3. GR activity was significantly higher in all inoculated roots compared to uninoculated counterparts across all soil levels with an increase of 46% and 68% in roots of plants cultivated in S2 and S3 and about 6-fold in plants cultivated in S1. Likewise, inoculation significantly enhanced SOD activity by 153% and 77% in roots of plant cultivated in S1 and S2, respectively. For CAT an enhanced activity was detected with inoculated roots demonstrating 2.5 and 4-fold higher activity in S1 and S2, respectively as compared to uninoculated plants. However, in the highly contaminated S3 soil, uninoculated roots exhibited the highest CAT activity, surpassing the inoculated ones (Table 3). For GPOX, no significant interaction was found, but both main effects were significant; inoculation increased GPOX activity across all soils, and S2 soil significantly stimulated this enzyme compared to S1 and S3. In contrast, root APX activity remained statistically stable, showing no significant variation regardless of the treatment or soil type.

In the aerial tissues, the enzymatic response followed a different pattern. Shoot SOD activity was significantly reduced by inoculation across all soil types, while it increased in uninoculated plants as a function of contamination levels. A significant interaction was also observed for shoot GR and APX. In uninoculated plants, APX activity was significantly increased only in shoots of plants cultivated in the soil S2 by 159%, whereas GR activity was significantly enhanced in shoots of plants cultivated in S2 and S3 (by approximately 6 and 4 times, respectively).

Contrariwise, GPOX activity was totally inhibited in uninoculated plants with increasing HMs contamination while inoculated plants maintained their GPOX activity in shoots of plants cultivated in S2 and S3 soils. Finally, CAT activity was significantly lower in inoculated plants from S1 and S3 (Table 3).

### Effects of *V. faba* L. *minor* var. Saber 02 inoculation by PGPB on plant heavy metals accumulation

**Fig 1:**
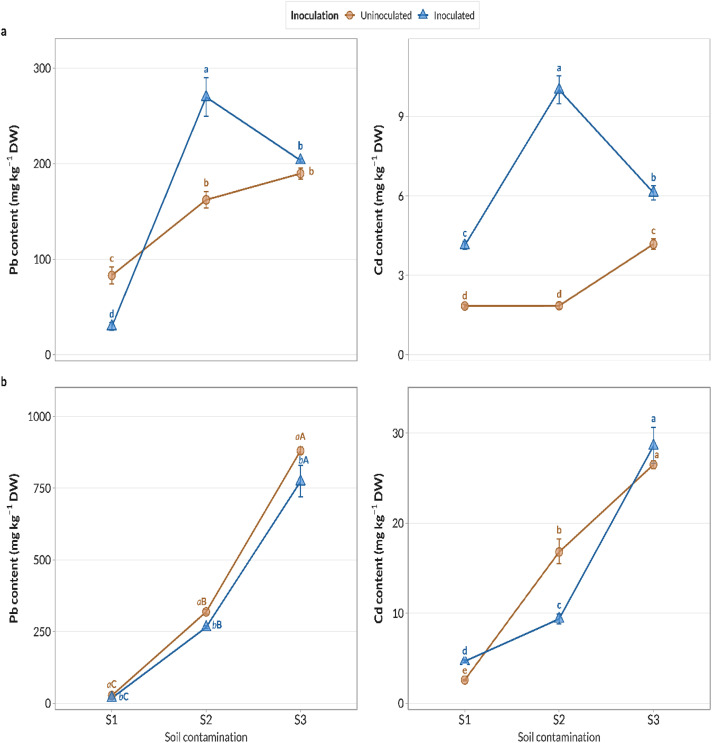
Effect of soil contamination and plant inoculation on heavy metal content in *V. faba* L. var. *minor* Saber 02. (a) Pb content (left) and Cd content (right) in shoots. (b) Pb content (left) and Cd content (right) in roots. Points represent mean ± SE (n = 3). Data were analysed by two-way ANOVA. When the IN × SC interaction was significant (p < 0.05), letters compare all treatment combinations by Tukey HSD (α = 0.05); letter colours match inoculation treatment. When the interaction was NS (p ≥ 0.05), the additive model was used; uppercase bold letters indicate Tukey HSD groupings for soil contamination; lowercase italic letters indicate groupings for inoculation treatment (α = 0.05).

Results revealed that inoculation significantly influenced the accumulation and tissue-specific distribution of Pb and Cd in the plants. Regarding shoot accumulation (Fig 1a), significant interactions between inoculation and soil contamination were observed for both Pb and Cd (Table 2). Inoculation enhanced shoot metal content, particularly in the moderately contaminated S2 soil, where Pb and Cd concentrations increased by 66% and 441%, respectively, compared to uninoculated plants. In contrast, the response in the roots (Fig 1b) followed a different pattern. For root Cd, a significant interaction showed that inoculation reduced metal accumulation by 44% in S2 soil. For root Pb, the interaction was non-significant; however, the additive model confirmed that while Pb levels rose sharply with increasing soil contamination, inoculation exerted a significant effect by slightly lowering the overall Pb concentration in the root system.

**Fig 2:**
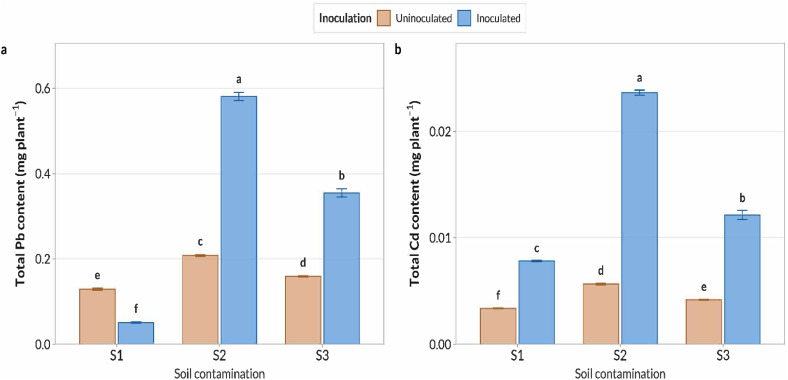
Effect of soil contamination and plant inoculation on total heavy metal content in *V. faba* L. var. *minor* Saber 02. (a) Total Pb content. (b) Total Cd content. Bars represent mean ± SE (n = 3). Means with a common letter are not significantly different at p < 0.05 according to Tukey’s HSD test.

Regarding the extraction capacity of the plants, expressed as total HM content per plant (Fig 2), a significant interaction between inoculation and soil contamination was observed both Pb and Cd. In fact, results demonstrated that *V. faba* L. var. *minor* Saber 02 extraction capacity was more pronounced under the moderately heavy metal contamination (S2) and to lesser degree in S3 soil. Likewise, in S2 soil, the inoculated *V. faba* L. var. *minor* Saber 02 extracted more HMs compared to uninoculated plants, where the accumulation of Pb and Cd was about 179% and 319% higher, respectively.

On the other hand, our analysis revealed that the translocation factors were significantly affected by the interaction IN x SC (Table 2). In fact, TF of Cd increased upon inoculation by 23% and 31% in plants grown in S1 and S3 respectively and about 10-fold in plants grown in S2, where it was greater than one (Table 4). Besides, inoculation significantly enhanced TF value of Pb in plants grown in moderately contaminated soil by up to 73% compared to the uninoculated ones.

**Table 4:** Effects of soil contamination and plant inoculation on HMs translocation factor in *V. faba.* L. var. *minor* Saber 02.

	Uninoculated			Inoculated		
	S1	S2	S3	S1	S2	S3
**Cd**	0.71 ± 0.015^c^	0.11 ± 0.003^f^	0.16 ± 0.009^e^	0.88 ± 0.006^b^	1.07 ± 0.009^a^	0.21 ± 0.01^d^
**Pb**	3.06 ± 0.1^a^	0.51 ± 0.024^d^	0.22 ± 0.006^e^	1.63 ± 0.21^b^	0.88 ± 0.12^c^	0.26 ± 0.03^de^

Data are means of three replicates. Within each line, means followed by a common letter are not significantly different at p < 0.05 according to Tukey’s HSD-test.

### Effects of *V. faba* L. var. *minor* Saber 02 inoculation by PGPB on total and metal available fractions and soil characteristics

In this part we evaluated the effects of *V. faba* L. var. *minor* Saber 02 cultivation on total and available fractions of Cd and Pb, as well as soil nitrogen and phosphorus content. In addition, some biochemical activities were investigated in the soil, which can improve plants growth under heavy metal stress.

Regarding soil fertility, a significant interaction was observed for total nitrogen. In uninoculated soils, nitrogen content declined progressively with increasing contamination, falling by 39% in S3 compared to the S1 control (Table 5). However, the use of the bacterial consortium significantly reversed this trend, enhancing total N by 30%, 55%, and 33% in S1, S2, and S3, respectively. For available phosphorus, only the independent effects of inoculation and soil contamination were significant. Inoculation significantly increased available P (approximately 32% across all soil levels), while P_2_O_5_ also rose progressively with increasing HM contamination, regardless of inoculation status.

**Table 5:** Effects of contamination and *Vicia faba* L. var. *minor* Saber 02 inoculation on soil characteristics.

			Uninoculated			Inoculated		
			S1	S2	S3	S1	S2	S3
**Total N** (%)			0.54 ± 0.02^b^	0.44 ± 0.04^c^	0.33 ± 0.01^d^	0.71 ± 0.03^a^	0.68 ± 0.01^a^	0.44 ± 0.04^c^
**P**_**2**_**O**_**5**_(ppm)			16.4 ± 1.8^**C***b*^	22.9 ± 0.3^**B***b*^	29.1 ± 1.2^**A***b*^	27.7 ± 0.1^**C***a*^	30.1 ± 2.5^**B***a*^	33.5 ± 4.6^**A***a*^
***β*-Glucosidase** (µg PNP g^-1^soil h^-1^)			40 ± 3.5^a^	42.3 ± 3.5^a^	26.5 ± 1.5^b^	37.4 ± 4.1^a^	39.4 ± 4.2^a^	40 ± 2.9^a^
**Urease** (µg N g^-1^ soil h^-1^)			58.5 ± 2.5^c^	44.4 ± 0.5^d^	43.1 ± 1.7^d^	72.7 ± 4.6^b^	52.3 ± 3.2 cd	97.9 ± 10.4^a^
**Heavy metal in the soil** (mg kg ^− 1^ DW)	Total	Cd	0.43 ± 0.03^d^	2.4 ± 0.2^b^	3.2 ± 0.2^a^	0.41 ± 0.03^d^	2.1 ± 0.14^c^	2.7 ± 0.15^b^
		Pb	7.3 ± 0.2^d^	96.9 ± 7.8^b^	295.8 ± 12.9^a^	5.5 ± 0.4^d^	70.84 ± 4.3^c^	283.6 ± 17.9^a^
	Extractable	Cd	0.05 ± 0.01^e^	0.92 ± 0.05^c^	2.65 ± 0.11^a^	0.06 ± 0.01^e^	0.74 ± 0.04^d^	1.25 ± 0.09^b^
		Pb	1.94 ± 0.1^d^	67.9 ± 0.5^b^	120.8 ± 1.4^a^	1.66 ± 0.4^d^	61.3 ± 2^c^	119.2 ± 0.2^a^

Data are means ±standard deviation (n = 3). Within each line, means followed by a common letter are not significantly different at *p* < 0.05 according to Tukey’s HSD-test.

The biochemical health of the soil was similarly improved by the presence of the PGPB. A significant interaction was noted for *β*-glucosidase activity; while heavy metal stress in S3 reduced this activity by 33% in uninoculated soils, inoculation successfully improved it to levels comparable to the S1 control. Urease activity also demonstrated a significant interaction. In uninoculated plants, heavy metal contamination significantly inhibited urease activity by approximately 26% at both S2 and S3. However, inoculation not only prevented this inhibition but reversed it, with the greatest stimulation observed in S3 (127% above uninoculated S3) (Table 5).

The analysis of soil heavy metal confirms that the plant-microbe association significantly influenced Pb and Cd levels. For both total and extractable fractions of Pb and Cd, a significant interaction was observed, indicating that the impact of inoculation was dependent on the level of soil contamination. Specifically, regarding total metal content, inoculation significantly reduced Total Pb in the moderately contaminated S2 soil by 27%, whereas no significant reduction was achieved in the highly contaminated S3 (Table 5). In contrast, Total Cd was significantly reduced across both contaminated treatments, decreasing by 13% in S2 and 16% in S3 upon inoculation. A similar trend was observed for the bioavailability of these metals. Inoculation significantly decreased the Extractable fraction of Cd in both S2 (by 20%) and S3 (by 53%) compared to uninoculated soils. For Extractable Pb, while the interaction was statistically significant, the most notable reduction occurred in the S2 soil, while concentrations in S3 remained statistically similar regardless of inoculation.

## Discussion

The cultivation of *V. faba* L. var *minor*. Saber 02 in three soil samples differently contaminated by HMs (Pb and Cd), shows a negative impact on plant growth, nodulation, chlorophyll, nitrogen and phosphorus content. It may be caused by reduction of water and nutrient uptake, since generally, HMs can interfere with uptake, transport and use of nutrient elements [44].

The inoculation with PGPB had a positive impact on plant growth parameters, owing to the amelioration of plant metabolism processes like symbiotic nitrogen fixation and photosynthesis. In fact, the used bacterial consortium was characterized by their nodulation capacities, HMs tolerance and production of plant growth promoting substances [20]. HMs tolerance of PGPB is an important factor which affects HMs tolerance of host plant and improves its potential for bioremediation, as they have the ability to promote the growth of the host plant by various mechanisms like nitrogen fixation, production of PGPs substances [9,13,16]. Many other PGPBs were characterized by their PGP traits such as those nodulating *Sulla coronaria* [45] and *Lathyrus sativus* [17], all these PGPs were not only effective on plant growth, but also essential to the legume-rhizobia symbiosis for effective nodulation and nitrogen ﬁxation [15].

The evaluation of plant lipid peroxidation through the generation of malondialdehyde demonstrated that HMs also induced a substantial increase in MDA in roots of *V. faba*. However, the inoculation decreased the harmful effects of oxidative damage caused by ROS production. The ameliorative effect of inoculation could be due to nodules which are rich in antioxidant that limit the production of reactive oxygen species [45,46].

The present research showed a significant increase in SOD, CAT and GR activities in response to HMs contamination of soil, mainly in the roots. Significant rise in enzyme activities has been depicted in various plants species facing heavy metal stress [24,47,48]. Kandziora-Ciupa et al. [49] demonstrated the relationship between the availability of metals in the soil (Cd, Fe, Mn, Pb and Zn), their concentrations in leaves of *Vaccinium myrtillus* L. and the antioxidant response of this plant. Indeed, enhanced level of SOD reduced the quantity of damaging superoxide anion radical [50], likewise, increases in CAT activity should be induced by excessive H_2_O_2_ production. In addition, GR is a key enzyme of the ASH-GSH cycle and is responsible for maintaining the supply of reduced glutathione that plays an important role in the defence system against ROS [48].

Results analysis also showed a positive regulation of enzyme activities in PGPB inoculated plants, which is in conformity with some other findings [24,51], suggesting that this effect may be associated with the alleviation of oxidative stress through PGPB-mediated regulation of detrimental ROS accumulation. Moreover, this could be due to the important role of nodules in HMs stress mitigation by their antioxidant enzymes. Shah et al. [4] reported that PGPB alleviated metal toxicity and improved growth attributes in *Solanum melongena* via the activation of antioxidative defence system. Likewise, the response of antioxidant enzymes to HMs varied with plant species, plant tissues, the metal concentration, period of treatment and bacterial inoculation.

Analyses of Pb and, Cd accumulations in *V. faba* L. var. *minor* Saber 02 demonstrated that their concentrations increased with the increasing concentration of heavy metals in soils, essentially in roots. These results were confirmed in other reports demonstrating that the uptake and translocation of metals from soils to plants was due to HMs bioavailability, concentrations, soil structure and water activity [10,52]. Previous studies have shown that soil physicochemical characteristics strongly influence the mobility, speciation, and bioavailability of heavy metals, which in turn affect their uptake by plants [53,54]. Soil pH is a key factor, as acidic conditions generally increase the solubility of metal cations by reducing their adsorption to mineral and organic surfaces, whereas neutral to alkaline conditions tend to promote metal precipitation and immobilization [55,56]. Soil organic matter also plays an important role in heavy metal dynamics. It can bind metal ions through complexation and adsorption, lowering the concentration of free metals in the soil solution and restricting plant uptake. At the same time, certain soluble organic-metal complexes may enhance metal mobility depending on soil properties and the quality of the organic matter [57,58]. Further, root exudates such as organic acids play an important role in phytoremediation by binding metal ions and changing metal mobility, solubility and bioavailability in soil inducing their precipitation and/or complexation [59].

Results revealed that PGPB inoculation of *V. faba* L. var. *minor* Saber 02 further enhanced HMs concentrations in shoots, suggesting that this symbiosis is a promising biological material for metal phytoremediation purposes moderately HMs contaminated soils. In general, the inoculated legumes extracted more HMs under moderate HMs pollution, since the extreme pollution affected essential plant functioning and metabolic processes like nodulation, mineral uptake, photosynthesis, respiration and enzymatic activities [11,15,18]. Increased metal concentrations in plant tissues after PGPB inoculation is in agreement with other studies [6,13,24]. Nevertheless, plant responses to HMs contamination depended on PGPB which enhances plant uptake of HMs in certain cases or reduce them in other cases [10,16]. Furthermore, *V. faba* L. var. *minor* Saber 02 extraction capacity was more pronounced under the moderately HMs pollution essentially after inoculation, which can be associated with increased plant biomass production and the soil metal content in the HMs translocation to the aerial parts of plants. Obtained results could be related to PGP traits of used strains which induces the amelioration of nodulation that improves the uptake of HMs due to their metal bioaccumulation ability and their crucial role in HMs uptake by nodulated plant [9,11,20]. However, increased metal translocation to the aerial parts may represent a potential risk, particularly when edible crops are involved [60]. The accumulation of heavy metals in aboveground tissues raises concerns regarding food safety and limits the direct use of crops such as *Vicia faba* for human or animal consumption under contaminated environments [61,62]. Therefore, although *V. faba* showed promising responses in terms of tolerance and growth promotion, its use should be carefully considered and preferably restricted to phytoremediation purposes rather than food production in contaminated soils [63]. Nevertheless, the safe management of post-phytoremediation contaminated biomass poses significant practical challenges, particularly with respect to preventing site recontamination and the potential transfer of contaminants to the food chain [64]. The harvested biomass can serve as a valuable resource of novel catalysts or reusable materials. Moreover, through gasification or pyrolysis of post-remediation biomass, bioenergy products can be used for heating and electricity generation [64,65].

In addition, PGPB have the ability to produce various metabolic compounds such as organic acids, exo-polysaccharide, siderophores and biosurfactants etc., which can affect the metal mobility and availability in the rhizosphere that helps in metal uptake therefore contributing directly to the phytoremediation processes [13,66].

Indeed, bacterial strains used in the present study have the ability to produce siderophore, which may have increased HMs mobility toward plants, resulting in enhancing metals uptake by inoculated *V. faba* L. var. *minor* Saber 02. Likewise, increased metal accumulation in inoculated plants may be attributed to the ability of the PGPB to synthesize indole acetic acid which indirectly improves plant growth and biomass translated into a greater metal uptake [12,67].

Analysis of nitrogen and phosphorus content, which are able to ameliorate plants growth under HMs stress, showed that total nitrogen significantly decreased with increasing soil contamination. The inoculation of *V. faba* L. var. *minor* Saber 02 enhanced total N for the three studied soils, these results suggested the role of the used PGPB as biofertilizer which enhanced N content in soil through their nitrogen fixation capacity [15]. Phosphorus content was more available when soil contamination increased. Furthermore, inoculation enhanced significantly available phosphorus content, which could be associated with the capacity of phosphorus solubilisation of PGPB as previously demonstrated [68]. It has been reported that insoluble phosphate in metal polluted rhizosphere soils is likely to become bio-available by the plant associated bacteria through their organic acid production and acid phosphatase secretion [69]. However, the enhancement of soil P_2_O_5_ at the same time with the soil contamination can be related to a deficiency in its assimilation by the plants cultivated in high contaminated soil, presenting an important decrease in their biomass production. In fact, phosphorus mineralization refers to the solubilisation of organic phosphorus and the degradation of high molecular weight organic phosphate to lower molecular one, these actions are affected by environmental conditions such as the plant and soil conditions, bacterial strains, the pH of the rhizosphere and, phosphorous availability as well as the phytase produced by plant roots [52,70].

The soil enzyme activities have been used as ecological indicators for diagnosing, soil fertility and soil quality because of their stability and sensitivity; they can well indicate whether the biochemical reactions in the soil to which they are involved are correctly performed [71–73]. Moreover, they could be used as indicators to diagnose pollution and soil remediation processes and monitor the remediation effectiveness for sustainable remediation [74,75].

HMs contamination reduced β-glucosidase and urease activities, consistent with prior studies [73,76,77]. Following PGPB inoculation, an increase in these enzyme activities was observed; however, this effect is currently associative, as microbial biomass or community structure data were not measured. Similar associative trends have been reported by Abdelkrim et al. [14] and Ju et al. [40]. Therefore, while PGPB inoculation is linked to enhanced enzyme activities under contamination stress, further studies incorporating microbial community analyses are needed to establish causality.

Therefore, the increase in soil enzyme activities as well as P and N content in HMs contaminated soils following bacterial inoculation suggests that *V. faba* L. var. *minor* Saber 02 -PGPB symbiosis is an effective association for sustainable remediation.

Another finding of our study is that inoculation significantly decreased total Pb in moderately contaminated soil as well as total and extractable Cd in both moderately and highly contaminated soils, respectively. This is in agreement with the findings of Abdelkrim et al. [14] who reported that the inoculation of certain legumes cultivated in HMs contaminated soil had modified the concentration of metals in the soil.

## Conclusions

The results of the current study demonstrate that soil HMs contamination has detrimental effects on growth and biomass production of *V. faba* L. var. *minor* Saber 02, besides the increased synthesis of MDA in plant roots. The interaction between inoculation and soil contamination level significantly influenced shoot and nodule biomass, with PGPB inoculation showing the greatest benefits in moderately contaminated soil. Moreover, bacterial inoculation mitigated heavy metal induced nitrogen depletion, with the greatest benefits in moderately contaminated soil. While stress reduced N in uninoculated plants, inoculated plants maintained higher shoot and root N even under severe contamination. These findings highlight PGPB’s potential to improve plant nitrogen nutrition under heavy metal stress.

In addition to improving growth and nitrogen nutrition, PGPB enhanced plant stress tolerance by modulating the antioxidant system, increasing the activity of SOD, CAT, and GR, particularly in roots.

Furthermore, the inoculation of *V. faba* L. var. *minor* Saber 02 cultivated in the moderately contaminated soil, enhanced Pb and Cd accumulation in shoots, indicating the role of symbiosis partner’s state and their tolerance in phytoremediation process.

Similarly, bacterial inoculation markedly reduced total Pb in moderately contaminated soil as well as the total and available fractions of Cd in both moderately and highly contaminated soils. Increasing levels of soil contamination were associated with significant declines in total nitrogen content and the enzymatic activities of β-glucosidase and urease. However, PGPB inoculation significantly mitigated these negative effects, enhancing both nitrogen availability and soil enzyme activities.

These findings indicate that PGPB function effectively as biofertilizers by enhancing nitrogen levels likely through biological nitrogen fixation and by stimulating key enzymatic functions critical for soil health.

Furthermore, PGPB may serve as essential components in sustainable agricultural systems, contributing to the remediation of moderately heavy metal (HM)-polluted soils and counteracting ecological disruptions, including the degradation of native soil microbiota and fauna. Additional investigations are necessary to elucidate the molecular mechanisms underpinning heavy metal tolerance within the *Vicia faba* L. var. *minor* Saber 02 PGPB symbiotic interaction.
